# Soft-tissue thickness changes following bimaxillary surgery in skeletal class II malocclusion

**DOI:** 10.1007/s00784-026-07074-5

**Published:** 2026-08-11

**Authors:** Hao Chen, Dirk-Melle Beek, Frank Baan, Stephan Christian Möhlhenrich, Kasper Stokbro, Tong Xi

**Affiliations:** 1 Department of Oral and Maxillofacial Surgery, School and Hospital of Stomatology, Guangdong Engineering Research Center of Oral Restoration and Reconstruction, Guangzhou Key Laboratory of Basic and Applied Research of Oral Regenerative Medicine, Guangzhou Medical University, Huangsha Avenue 39, Liwan District, Guangzhou, Guangdong 510150 China; 2 Department of Dentistry, Section of Orthodontics, Dentofacial Orthopedics and Craniofacial Biology, Radboud University Medical Center, P.O. Box 910, 309, Nijmegen, HB 6500 The Netherlands; 3 Department of Oral and Maxillofacial Surgery, Radboud University Medical Center, PO Box 9101, Nijmegen, 6500 the Netherlands; 4 Radboudumc 3D Lab, Radboud University Medical Center, Geert Grooteplein 10, Nijmegen, HB 6500 Netherlands; 5 Department of Oral and Maxillofacial Surgery, Odense University Hospital, Sdr. Boulevard 29, Odense, 5000 Denmark; 6 Department of Maxillofacial Research, Clinical institute, Faculty of Health, University of Southern, Sdr. Boulevard 29, Odense, 5000 Denmark

**Keywords:** Orthognathic surgery, Malocclusion, Angle class II, Cone-beam computed tomography, Soft-tissue thickness, Facial esthetics.

## Abstract

**Objectives:**

This retrospective cohort study evaluated regional facial soft-tissue thickness changes after bimaxillary surgery in skeletal Class Ⅱ patients and assessed their associations with skeletal movement and patient-related factors.

**Materials and methods:**

Class Ⅱ patients treated with bimaxillary advancement surgery without genioplasty were included. Postoperative CBCT scans, acquired 16.7 ± 5.3 months after surgery, were registered to the corresponding preoperative scans using voxel-based superimposition. Skeletal displacement and corresponding overlying soft-tissue thickness were assessed at subspinale (A), supradentale (Sd), infradentale (Id), supramentale (B), pogonion (Pog), and menton (Me). Soft-tissue thickness was defined as the linear distance between each hard-tissue landmark and its corresponding soft-tissue landmark. Skeletal displacement was measured at the corresponding hard-tissue landmarks.

**Results:**

89 patients were enrolled, 33 male and 56 female, mean age of 30.3 ± 10.3 years. Significant postoperative soft-tissue thinning was observed at A (0.40 ± 1.51 mm), Id (2.20 ± 2.11 mm), B (0.33 ± 1.30 mm), and Pog (0.48 ± 1.29 mm), whereas soft-tissue thickness at Sd and Me did not change significantly. Pearson correlation analysis indicated that thickness changes were not associated with age, sex, or CBCT interval. Larger sagittal advancement at A and Id was associated with more pronounced thinning of the corresponding soft tissue (A: *r* = -0.650, *p* < 0.001; Id: *r* = -0.338, *p* = 0.001), with ratios of thinning to movement of 0.20 and 0.30, respectively. In contrast, larger downward vertical movement of B was associated with less thinning at B (*r* = 0.240, *p* = 0.023), with a ratio of thinning to downward movement of 0.05.

**Conclusion:**

Bimaxillary surgery in skeletal Class II patients without genioplasty resulted in region specific soft-tissue thinning, particularly affecting the lower lip.

**Clinical relevance:**

Postoperative soft-tissue thinning should be considered during surgical planning and patient communication.

**Supplementary Information:**

The online version contains supplementary material available at https://doi.org/10.1007/s00784-026-07074-5.

## Introduction

Skeletal Class II malocclusion is commonly characterized by mandibular hypoplasia, increased overjet, a convex facial profile, chin retrusion or a weak chin, and acute labiomental fold [1]. Although orthognathic surgery is able to correct the skeletal relationship and can establish a functional occlusion, the final facial appearance depends on the indirect and complex response of the overlying soft tissues [2, 3]. Prediction of postoperative soft-tissue outcomes remains challenging because skeletal movement, muscular adaptation, orthodontic finishing, and soft-tissue tension interact in a region-specific manner [4–6]. Therefore, accurate assessment of postoperative soft-tissue changes is essential for evaluating facial harmony and treatment outcomes in skeletal Class II patients.

Previous studies have mainly focused on soft-tissue landmark displacement, profile changes, and soft-to-hard tissue movement ratios after orthognathic surgery. In skeletal Class II patients treated with BSSRO advancement alone, Storms et al. reported close hard-to-soft tissue correspondence in the chin region, whereas lower-lip movement represented only 76% of the corresponding hard-tissue movement [7]. Similarly, Bral et al. reported that, without genioplasty, the soft-tissue/bony pogonion (Pog) movement ratio was 87% after mandibular advancement alone and 102% after mandibular advancement combined with maxillary osteotomy, while the soft-tissue/bony menton (Me) ratio ranged from 85% to 96% depending on the direction of vertical movement [8]. More recently, three-dimensional analysis in high-angle skeletal Class II patients treated with bimaxillary surgery and genioplasty showed that most nasal and lip landmarks moved forward and upward, with sagittal soft-to-hard tissue ratios in the lower facial region ranging from 0.92 to 1.17 [9]. Thus, postoperative soft-tissue landmark changes are region specific and cannot be fully extrapolated from skeletal movements alone.

However, soft-tissue landmark displacement does not necessarily reflect changes in the thickness of the soft-tissue envelope. This distinction is clinically relevant, as regional soft-tissue thickness contributes to facial contour, lip support, chin prominence, and lower facial balance, all of which are important factors influencing facial esthetics [10–12]. Although CBCT is primarily designed for hard-tissue imaging, the linear distance between corresponding hard- and soft-tissue landmarks can be used to assess regional soft-tissue thickness when standardized anatomical landmarks are applied [13, 14]. Previous CBCT-based studies have investigated postoperative soft-tissue thickness changes after orthognathic surgery, mainly in skeletal Class III patients, mandibular setback, facial asymmetry correction, or adjunctive genioplasty [15–17]. Evidence regarding postoperative regional soft-tissue thickness changes in skeletal Class II patients remains limited. Therefore, this study aimed to evaluate postoperative thickness changes in upper and lower facial regions in skeletal Class II patients using paired preoperative and postoperative CBCT data, and to analyze their associations with skeletal movement parameters and patient-related factors.

## Materials and methods

### Study design and patients

This retrospective cohort study was conducted at the Department of Oral and Maxillofacial Surgery, Radboud University Medical Center, Nijmegen, The Netherlands. Consecutive patients with skeletal Class II malocclusion who underwent bimaxillary orthognathic surgery between January 2016 and December 2025 were screened for eligibility. The study was approved by the regional medical ethics review board of Radboudumc Nijmegen (registration number 2026–18955), and informed consent was obtained from all participants. All data were anonymized and de-identified before analysis, and the use of clinical data followed the conditions specified in the ethical approval.

The inclusion criteria were: skeletal Class II malocclusion treated with bimaxillary surgery consisting of Le Fort I osteotomy and bilateral sagittal split osteotomy; age of 16 years or older at the time of surgery; availability of preoperative (T0) and postoperative (T1) CBCT scans with adequate image quality for three-dimensional analysis; and a minimum interval of 6 months between surgery and the postoperative CBCT scan. Patients were excluded if they had craniofacial syndromes, cleft lip and/or palate, previous orthognathic surgery, maxillofacial trauma, congenital dental anomalies, incomplete imaging of the regions of interest, severe motion artefacts, or genioplasty performed before or during the bimaxillary procedure.

Demographic and clinical variables, including age, sex, body mass index (BMI), surgical date, and the interval between the preoperative and postoperative CBCT scans, were retrieved from the clinical records.

### Surgical procedure

All surgeries were performed by the same experienced oral and maxillofacial surgeon (T.X.) using a standardized surgical protocol. No substantial changes in surgical planning, operative technique, fixation protocol, or perioperative management occurred during the study period. All bimaxillary procedures were performed under general anaesthesia with nasotracheal intubation according to the preoperative virtual surgical plan. The maxilla was mobilized using a conventional Le Fort I osteotomy, which allowed three-dimensional repositioning of the dentoalveolar maxillary segment. After down-fracture and mobilization, the maxilla was positioned with a prefabricated intermediate occlusal splint and stabilized with four titanium miniplates and monocortical screws (KLS Martin, Tuttlingen, Germany and Stryker, Kalamazoo, MI, USA), one paranasal and one at the maxillary buttress on each side.

The mandibular procedure was performed as a bilateral sagittal split osteotomy according to Hunsuck modification [18]. After completion of the osteotomies, the distal mandibular segment was positioned using the final occlusal splint and stabilized with intermaxillary fixation. The proximal segments were seated carefully to position the condyles in the glenoid fossae. Rigid internal fixation of the mandible was performed using one titanium miniplate and monocortical screws on each side (KLS Martin, Tuttlingen, Germany and Stryker, Kalamazoo, MI, USA). After release of intermaxillary fixation, the occlusion was checked and tight elastics were applied.

### CBCT analysis and measurements

**Fig. 1:**
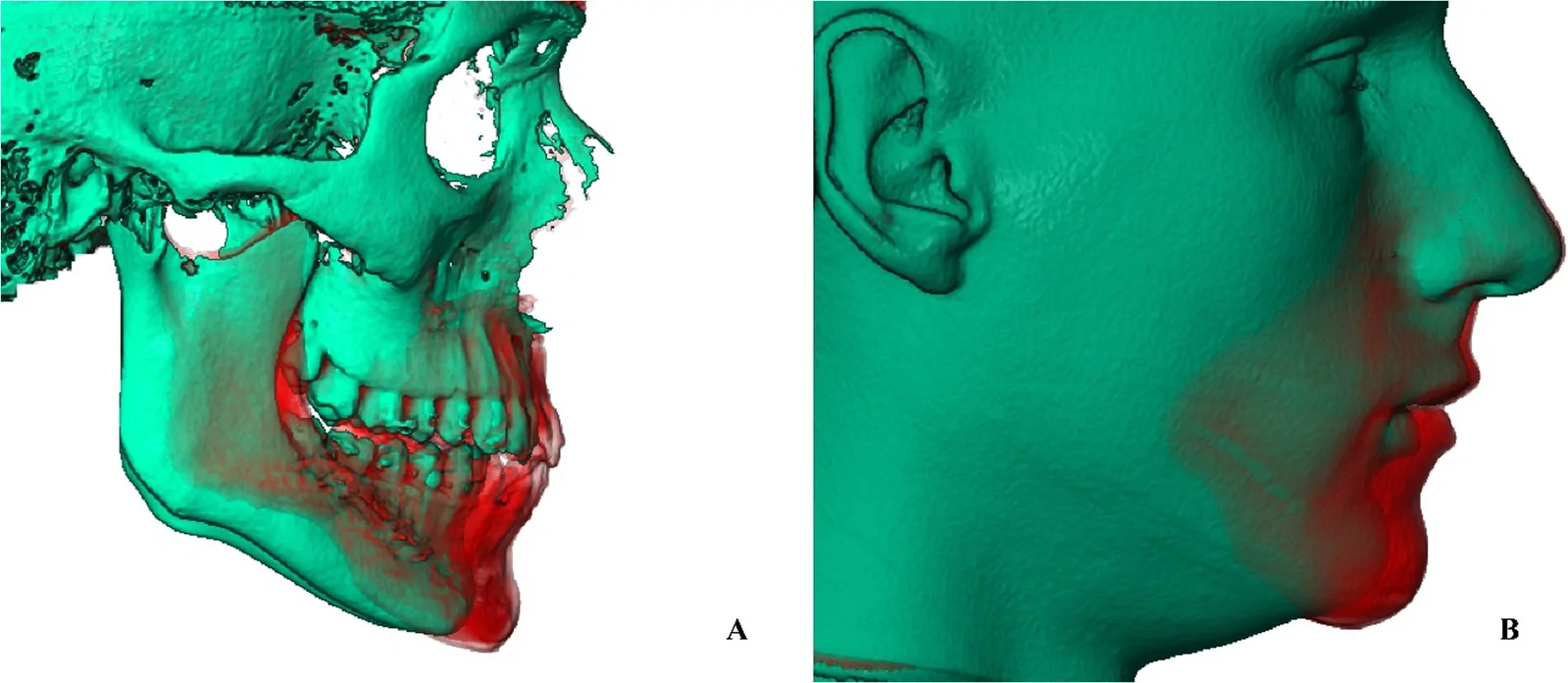
3D superimposition of T0 and T1 CBCT-derived skeletal and facial soft-tissue models. (**A**) Skeletal superimposition after voxel-based registration, with the T1 CBCT model displayed transparently to visualize skeletal changes between time points. (**B**) Facial soft-tissue surface superimposition, with the T1 soft-tissue model displayed transparently to illustrate postsurgical soft-tissue changes

For each included patient, the preoperative CBCT scan used for surgical planning was identified as T0, and an available postoperative CBCT scan obtained at least 6 months after surgery was identified as T1. DICOM datasets were imported into 3DMedX^®^ software (v1.2.46.0, 3D Lab Radboudumc, Nijmegen, The Netherlands) for 3D analysis. Before voxel-based registration, a patient-specific 3D reference system was constructed using the Frankfurt horizontal plane, sella coronal plane, and midsagittal plane. The T1 CBCT was then registered to the T0 CBCT by voxel-based superimposition of the cranial base (Fig. 1) [19]. After registration, the same reference system was shared by the registered T0 and T1 datasets.

**Fig. 2:**
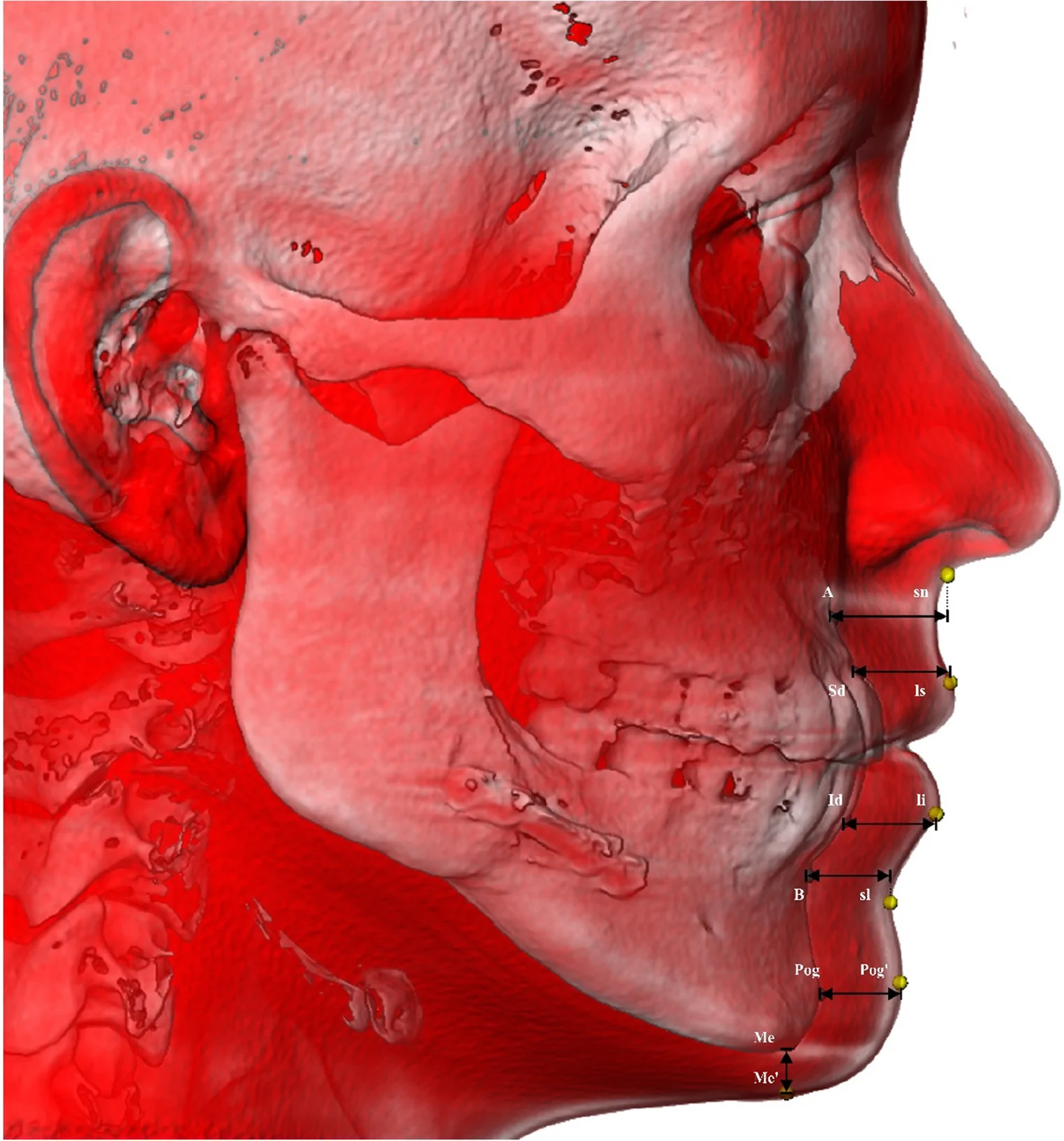
Measurement of regional soft-tissue thickness on a representative three-dimensional CBCT reconstruction at T1. Hard- and soft-tissue landmarks were identified on the three-dimensional reconstructed model. Horizontal reference lines, parallel to the horizontal axis, were constructed through the selected hard-tissue landmarks on the midsagittal plane. Soft-tissue thickness was defined as the linear anteroposterior distance from each hard-tissue landmark to the corresponding soft-tissue landmark or its vertical projection. Me–Me′ was measured as the vertical linear distance between Me and Me′

Following previously published CBCT-based methods for soft-tissue assessment [14, 17], the soft-tissue outcome variables consisted of linear hard-to-soft tissue thickness measurements rather than volumetric assessments. The reference planes and anatomical landmarks used in this study are defined in Table 1. Baseline skeletal morphology, surgical skeletal movements, soft-tissue thickness, and profile angles were measured as described in Table 2. Hard- and soft-tissue landmarks were identified on the 3D reconstructed CBCT images. Soft-tissue thickness was defined as the linear distance between corresponding hard-tissue and soft-tissue landmarks in the specified facial region on the midsagittal plane (Fig. 2). Thickness change was calculated as T1 minus T0. Positive values indicated postoperative thickening and negative values indicated postoperative thinning of the corresponding soft-tissue region. The ratio of soft tissue thickness change to skeletal sagittal advancement was calculated as the mean soft-tissue thickness change divided by the mean corresponding skeletal sagittal advancement in the whole cohort.

**Table 1:** Definitions of landmarks and reference planes

Landmark/reference plane	Definition
Infraorbital point (Or)	The lowest point on the infraorbital rim of the orbit.
Porion (Po)	The upper margin of the external auditory canal.
Sella turcica (S)	The midpoint of the sella turcica of the sphenoid bone.
Nasion (N)	The most anterior point of the frontonasal suture.
Frankfurt horizontal plane (FHP)	The horizontal reference plane passing through bilateral porion and orbitale landmarks.
Sella coronal plane (SCP)	A coronal reference plane passing through sella and perpendicular to the Frankfurt horizontal plane.
Mid-sagittal plane (MSP)	A vertical midline plane passing through nasion and sella, perpendicular to the Frankfurt horizontal plane.
A-point (A)	The point of maximum concavity in the midline of the dentoalveolar process of the maxilla.
Subnasale (sn)	The midpoint on the nasolabial soft-tissue contour between the columella crest and the upper lip.
Supradentale (Sd)	The most superior point of the alveolar ridge between the maxillary central incisors.
Labrale superius (ls)	The midpoint of the vermilion border of the upper lip.
Infradentale (Id)	The most superior point of the alveolar ridge between the mandibular central incisors.
Labrale inferius (li)	The midpoint of the vermilion border of the lower lip.
B-point (B)	The point of maximum concavity in the midline of the dentoalveolar process of the mandible.
Sublabiale (sl)	The most posterior midpoint on the labiomental soft-tissue contour, defining the border between the lower lip and the chin.
Pogonion (Pog)	The most anterior midpoint of the chin on the outline of the mandibular symphysis.
Soft-tissue pogonion (Pog’)	The most anterior midpoint of the soft-tissue chin.
Menton (Me)	The most inferior midline point on the mandibular symphysis.
Soft-tissue menton (Me’)	The most inferior midpoint on the soft-tissue contour of the chin, located at the level of the hard-tissue menton landmark.

**Table 2:** Definitions of measurements

Measurement	Definition
SNA angle (degree)	The angle between the S-N line and the N-A line, projected onto the midsagittal plane at T0.
SNB angle (degree)	The angle between the S-N line and the N-B line, projected onto the midsagittal plane at T0.
ANB angle (degree)	The angle between the N-A line and the N-B line, projected onto the midsagittal plane at T0.
A movement (mm)	The change in point A between T0 and T1 in the transverse direction relative to the MSP, sagittal direction relative to the SCP, and vertical direction relative to the FHP.
B movement (mm)	The change in point B between T0 and T1 in the transverse direction relative to the MSP, sagittal direction relative to the SCP, and vertical direction relative to the FHP.
Pog movement (mm)	The change in Pog between T0 and T1 in the transverse direction relative to the MSP, sagittal direction relative to the SCP, and vertical direction relative to the FHP.
Me movement (mm)	The change in Me between T0 and T1 in the transverse direction relative to the MSP, sagittal direction relative to the SCP, and vertical direction relative to the FHP.
A-sn thickness (mm)	The horizontal distance from point A to subnasale measured on the midsagittal plane at T0 and T1.
Sd-ls thickness (mm)	The horizontal distance from supradentale to labrale superius measured on the midsagittal plane at T0 and T1.
Id-li thickness (mm)	The horizontal distance from infradentale to labrale inferius measured on the midsagittal plane at T0 and T1.
B-sl thickness (mm)	The horizontal distance from point B to sublabiale measured on the midsagittal plane at T0 and T1.
Pog-Pog’ thickness (mm)	The horizontal distance from hard-tissue pogonion to soft-tissue pogonion measured on the midsagittal plane at T0 and T1.
Me-Me’ thickness (mm)	The vertical distance from hard-tissue menton to soft-tissue menton measured on the midsagittal plane at T0 and T1.
Labiomental angle (degree)	The angle formed by the line connecting labrale inferius to sublabiale and the line connecting sublabiale to soft-tissue pogonion, measured on the midsagittal soft-tissue profile at T0 and T1.
Nasolabial angle (degree)	The angle formed by the columella tangent and the line connecting subnasale to labrale superius, measured on the midsagittal soft-tissue profile at T0 and T1.

### Statistical analysis

A priori sample size estimation was performed using G*Power software (version 3.1.9.7; Düsseldorf, Germany) for a paired-sample t test. The calculation was based on the soft-tissue thickness change reported by Kim et al. [17]. With a two-tailed alpha level of 0.05, a statistical power of 90%, and an estimated effect size of *dz* = 0.365, the minimum required sample size was 81 patients.

Statistical analyses were performed using SPSS software (IBM Corp., Armonk, NY, USA). Descriptive statistics were calculated for demographic variables, baseline skeletal characteristics, skeletal movements, soft-tissue thickness measurements, and profile angles. Continuous variables were expressed as mean and standard deviation, and categorical variables were expressed as numbers and percentages. To assess intra-observer reliability, measurements were repeated by the same observer in 20 randomly selected cases after a two-week interval. Relative measurement reliability was evaluated using the intraclass correlation coefficient (ICC) based on a two-way random effects model with absolute agreement for single measurements. The absolute measurement error of each variable, the duplicate measurement error (DME) was calculated from the corresponding repeated measurements using Dahlberg’s formula, DME = √(Σd²/2n), where d is the difference between the two measurements of each variable and n is the number of duplicated cases.

The normality of continuous variables was assessed using the Shapiro-Wilk test before parametric analyses. Paired t-tests were used to compare preoperative and postoperative soft-tissue thickness measurements, profile angles, and skeletal landmark positions. Pearson correlation analysis was used to assess associations between soft-tissue thickness changes and patient-related or skeletal movement variables. Age, sex, BMI, and CBCT interval were evaluated as patient-related variables. Skeletal movement variables included transverse, sagittal, and vertical movements of point A, Sd, Id, B, Pog and Me. All tests were two-sided, and the level of statistical significance was set at *p* < 0.05.

## Results

### Study sample

The study sample comprised 89 patients. Table 3 summarizes the baseline characteristics of all patients with complete baseline records, including 56 female patients (62.9%) and 33 male patients (37.1%). The mean age at surgery was 30.3 ± 10.3 years (range 16–61 years), and the mean BMI was 24.28 ± 4.23 kg/m2 (range 16.87 to 39.16 kg/m2). Baseline cephalometric measurements confirmed a skeletal Class II malocclusion, with mean SNA, SNB, and ANB angles of 80.63 ± 3.41 degrees, 74.95 ± 3.64 degrees, and 5.68 ± 2.78 degrees, respectively. The mean interval between the preoperative and postoperative CBCT scans was 16.7 ± 5.3 months (Table 3). The mean ICC was 0.98 ± 0.04, with values ranging from 0.87 to 0.99, indicating high intra-observer reliability of the measurements. The DME of the soft-tissue thickness measurements ranged from 0.14 to 0.35 mm, and that of angular measurements was ± 1 degree (Supplementary Table 1). These results support the consistency of landmark identification and suggest that the applied measurement protocol is sufficiently reliable to detect small changes in soft-tissue thickness at the cohort level.

**Table 3:** Baseline characteristics of the study sample (*n* = 89)

Variable	Unit	Value
Age	Years	30.3 ± 10.3
Gender		
Female	n (%)	56 (62.9%)
Male	n (%)	33 (37.1%)
BMI	kg/m^2^	24.28 ± 4.23
SNA	Degrees	80.63 ± 3.41
SNB	Degrees	74.95 ± 3.64
ANB	Degrees	5.68 ± 2.78
CBCT Interval	Months	16.7 ± 5.3

*BMI* body mass index. CBCT interval, months between preoperative (T0) and follow-up (T1) CBCT scans

### Skeletal movements

Skeletal movements between T0 and T1 are summarized in Table 5. In the maxillary region, point A showed significant sagittal advancement (2.01 ± 1.62 mm; *p* < 0.001) and upward movement (0.82 ± 2.28 mm; *p* = 0.001), whereas its transverse change was not significant. Sd also showed significant sagittal advancement (2.06 ± 1.70 mm; *p* < 0.001), with no significant transverse or vertical change.

Mandibular landmarks showed larger sagittal advancement than the maxillary landmarks. Point B, Pog, Me, and Id advanced by 7.19 ± 3.91 mm, 7.29 ± 4.34 mm, 7.53 ± 4.53 mm, and 7.36 ± 3.89 mm, respectively (all *p* < 0.001). Significant transverse positional changes were observed at point B (0.71 ± 2.41 mm; *p* = 0.007), Pog (0.78 ± 2.88 mm; *p* = 0.013), Me (0.73 ± 2.94 mm; *p* = 0.022), and Id (0.71 ± 2.35 mm; *p* = 0.006). In the vertical direction, only Me showed a significant downward movement (0.70 ± 2.67 mm; *p* = 0.016) among the mandibular landmarks, whereas point B, Pog, and Id showed no significant vertical changes.

### Soft-tissue thickness and profile angles

**Fig. 3:**
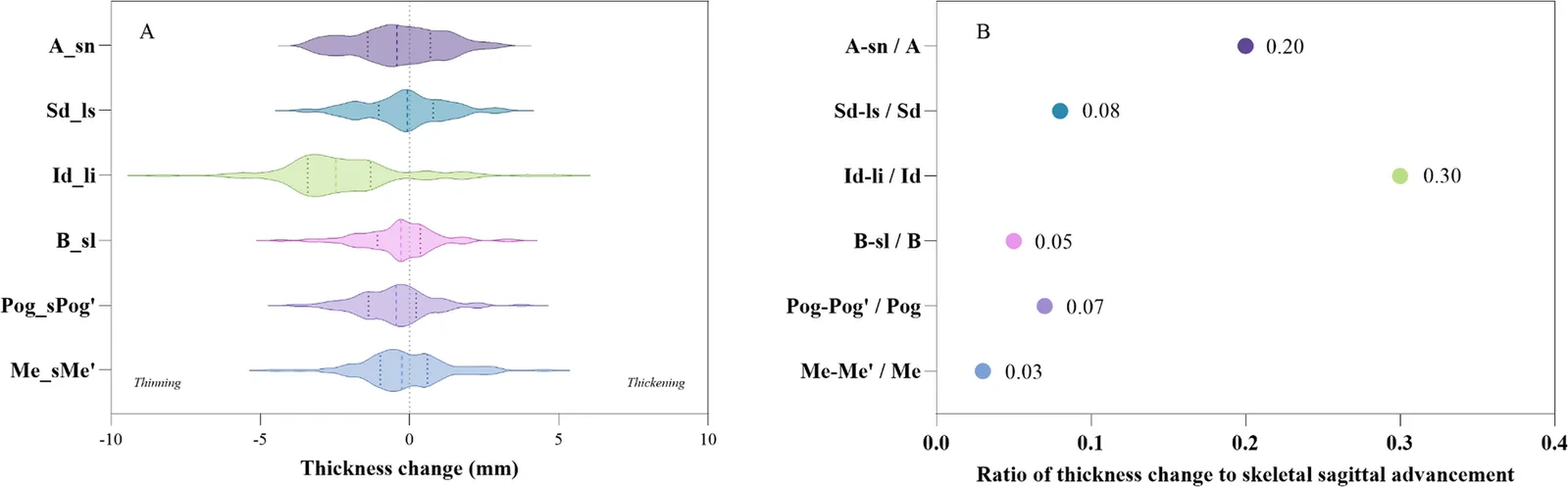
Regional facial soft-tissue thickness changes and ratios of thickness change to skeletal sagittal advancement after bimaxillary surgery. (**a**) Distribution of regional facial soft-tissue thickness changes after bimaxillary surgery. Positive values indicate postoperative thickening, whereas negative values indicate thinning. Each violin represents the distribution of individual observations; the central line indicates the median, and the adjacent lines indicate the interquartile range. (**b**) Regional ratios of soft-tissue thickness change to skeletal sagittal advancement. Higher values indicate a more soft-tissue thinning relative to skeletal advancement

Regional soft-tissue thickness changes and profile angle changes are presented in Table 4; Fig. 3A. Significant postoperative thinning was observed at A, Id, B, and Pog, with their corresponding soft-tissue landmarks. The largest regional change occurred at Id-li (2.20 ± 2.11 mm; *p* < 0.001), indicating pronounced thinning of the lower lip region. No significant thickness change was found at Sd-ls or Me-Me’. The labiomental angle increased significantly by 23.43 ± 16.73 degrees (*p* < 0.001), whereas the nasolabial angle showed no significant change, with a mean increase of 0.24 ± 6.44 degrees (*p* = 0.726) Table 5.

**Table 4:** Changes in soft-tissue thickness and profile angles between T0 and T1

Variable	T0	T1	Δ (T1-T0)	95% CI		*p*
				Lower	Upper	
A_sn (mm)	15.30 ± 2.50	14.90 ± 2.84	−0.40 ± 1.51	−0.72	−0.08	0.014^*^
Sd_ls (mm)	13.10 ± 2.38	12.94 ± 2.41	−0.17 ± 1.40	−0.46	0.13	0.260
Id_li (mm)	16.20 ± 2.50	14.00 ± 1.98	−2.20 ± 2.11	−2.65	−1.76	< 0.001^***^
B_sl (mm)	11.62 ± 1.81	11.29 ± 1.56	−0.33 ± 1.30	−0.61	−0.06	0.018^*^
Pog_Pog’ (mm)	13.20 ± 1.97	12,72 ± 2.04	−0.48 ± 1.29	−0.75	−0.20	< 0.001^***^
Me_Me’ (mm)	7.63 ± 2.54	7.42 ± 2.20	−0.20 ± 1.38	−0.50	0.09	0.165
LMA (degree)	109.73 ± 20.62	133.16 ± 11.40	23.43 ± 16.73	19.90	26.95	< 0.001^***^
NLA (degree)	113.50 ± 11.40	113.74 ± 10.70	0.24 ± 6.44	−1.12	1.60	0.726

Positive values for mean difference indicate postoperative thickening for linear soft-tissue thickness measurements; negative values indicate thinning. For angular variables, positive values indicate a postoperative increase, and negative values indicate a postoperative decrease. Paired t-tests were used for the primary analysis of paired changes. * *p* < 0.05, *** *p* < 0.001

**Table 5:** Three-dimensional skeletal landmark movements between T0 and T1

Landmark	Direction	Mean Difference	SD	95% CI		*p*
				Lower	Upper	
A	Transverse	0.01	1.12	−0.23	0.25	0.925
	Sagittal	2.01	1.62	1.67	2.35	< 0.001^***^
	Vertical	0.82	2.28	0.34	1.30	0.001^**^
Sd	Transverse	−0.07	1.13	−0.31	0.17	0.548
	Sagittal	2.06	1.70	1.70	2.42	< 0.001^**^
	Vertical	0.05	2.63	−0.51	0.60	0.865
Id	Transverse	0.71	2.35	0.21	1.20	0.006^**^
	Sagittal	7.36	3.89	6.54	8.18	< 0.001^***^
	Vertical	−0.24	3.21	−0.92	0.44	0.488
B	Transverse	0.71	2.41	0.20	1.21	0.007^**^
	Sagittal	7.19	3.91	6.37	8.01	< 0.001^***^
	Vertical	−0.50	3.51	−1.24	0.23	0.179
Pog	Transverse	0.78	2.88	0.17	1.39	0.013^*^
	Sagittal	7.29	4.34	6.38	8.21	< 0.001^***^
	Vertical	−0.32	2.99	−0.95	0.31	0.314
Me	Transverse	0.73	2.94	0.11	1.35	0.022^*^
	Sagittal	7.53	4.53	6.58	8.49	< 0.001^***^
	Vertical	−0.70	2.67	−1.26	−0.13	0.016*

Positive transverse values indicate movement toward the midline, and negative values indicate a shift from the midline. Positive sagittal values indicate advancement from T0 to T1, and negative values indicate setback. Positive vertical values indicate superior movement, and negative values indicate downward movement. Paired t-tests were used for the primary analysis of paired changes. * *p* < 0.05, ** *p* < 0.01, *** *p* < 0.001

### Correlation analysis

Pearson correlation analyses are summarized in Tables 6, 7, 8. No significant correlations were found between age, sex, or CBCT interval and regional soft-tissue thickness changes (Table 6). A larger Me-Me’ thickness change was negatively correlated with higher BMI (r = −0.281, *p* = 0.008), indicating more soft-tissue thinning at Me-Me’ in patients with higher BMI.

**Table 6:** Pearson correlations between demographic variables and regional soft-tissue thickness change (T1-T0)

Variable	Age		Sex		BMI		CBCT interval	
	Pearson *r*	*p*	Pearson *r*	*p*	Pearson *r*	*p*	Pearson *r*	*p*
A-sn	−0.136	0.202	−0.144	0.178	−0.106	0.323	0.057	0.598
Sd-ls	−0.003	0.975	0.017	0.887	−0.017	0.871	0.064	0.554
Id-li	−0.072	0.501	0.047	0.663	−0.089	0.407	−0.054	0.614
B-sl	−0.048	0.657	0.106	0.321	0.067	0.536	0.045	0.677
Pog-Pog’	0.084	0.434	−0.027	0.803	0.047	0.663	0.066	0.538
Me-Me’	0.004	0.973	0.127	0.237	−0.281	**0.008** ^**^	0.121	0.258

*BMI* body mass index. Positive correlations indicate less soft-tissue thinning or relative soft-tissue thickening; Negative correlations indicate more postoperative soft-tissue thinning. ** *p* < 0.01

**Table 7:** Pearson correlations between the regional soft-tissue thickness change (T1-T0) and the amount of skeletal Sagittal advancement

Variable	A		Sd		Id		B		Pog		Me	
	Pearson *r*	*p*	Pearson *r*	*p*	Pearson *r*	*p*	Pearson *r*	*p*	Pearson *r*	*p*	Pearson *r*	*p*
A-sn	**−0.650**	< 0.001***	**−0.355**	< 0.001***	0.114	0.288	0.182	0.088	**0.217**	0.041*	0.178	0.096
Sd-ls	**−0.244**	0.021*	**−0.325**	0.002**	0.032	0.768	0.065	0.548	0.102	0.341	0.073	0.495
Id-li	−0.021	0.842	0.031	0.775	**−0.338**	0.001**	−0.180	0.091	−0.120	0.265	−0.142	0.185
B-sl	−0.093	0.386	−0.025	0.817	−0.017	0.872	−0.035	0.744	−0.027	0.800	−0.064	0.551
Pog-Pog’	−0.013	0.904	0.047	0.664	0.031	0.770	0.018	0.867	−0.087	0.417	−0.097	0.364
Me-Me’	−0.018	0.865	−0.134	0.209	−0.032	0.767	−0.043	0.692	−0.055	0.606	−0.084	0.433

Positive correlations indicate that larger skeletal advancement was associated with less soft-tissue thinning or relative soft-tissue thickening, whereas negative correlations indicate that larger skeletal advancement was associated with more soft-tissue thinning

**Table 8:** Pearson correlations between the regional soft-tissue thickness change (T1-T0) and the amount of downward vertical skeletal movement

Variable	A		Sd		Id		B		Pog		Me	
	Pearson *r*	*p*	Pearson *r*	*p*	Pearson *r*	*p*	Pearson *r*	*p*	Pearson *r*	*p*	Pearson *r*	*p*
A-sn	−0.191	0.072	−0.192	0.072	0.095	0.373	0.066	0.540	0.072	0.501	0.092	0.393
Sd-ls	−0.009	0.933	−0.141	0.188	0.073	0.499	0.141	0.186	−0.046	0.667	−0.012	0.914
Id-li	0.168	0.115	−0.031	0.770	0.007	0.951	−0.097	0.368	−0.144	0.179	−0.131	0.222
B-sl	0.095	0.374	0.059	0.583	**0.243**	0.022*	**0.240**	0.023*	0.157	0.141	0.172	0.107
Pog-Pog’	0.029	0.786	0.091	0.396	0.058	0.592	0.035	0.743	0.031	0.776	−0.083	0.439
Me-Me’	−0.208	0.050	−0.174	0.103	**−0.291**	0.006**	**−0.288**	0.006**	**−0.251**	0.018*	**−0.357**	< 0.001**

Positive correlations indicate that larger downward skeletal movement was associated with less soft-tissue thinning or relative soft-tissue thickening, whereas negative correlations indicate that larger skeletal downward skeletal movement was associated with more soft-tissue thinning

**Fig. 4:**
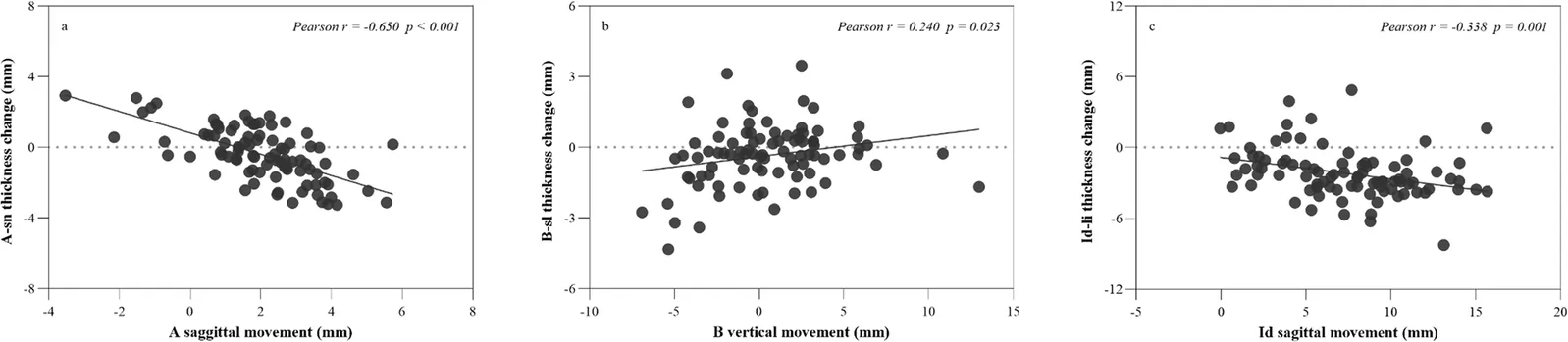
Correlations between skeletal movement amount and regional soft-tissue thickness change. Scatter plots showing significant correlations between skeletal movement amount (T1-T0) and regional soft-tissue thickness change (T1-T0). (**a**) Larger sagittal advancement of A point was associated with more A-sn soft-tissue thinning. (**b**) Larger downward vertical movement of B point was associated with less B-sl soft-tissue thinning. (**c**) Larger sagittal advancement of Id was associated with more Id-li soft-tissue thinning

For sagittal skeletal movements (Table 7), A-sn thickness change was negatively correlated with A-point and Sd sagittal advancement (*r* = −0.650 and − 0.355, respectively; both *p* < 0.001). Sd-ls thickness change was negatively correlated with A-point and Sd sagittal advancement (*r* = −0.244, *p* = 0.021; and *r* = −0.325, *p* = 0.002, respectively). Id-li thickness change was negatively correlated with Id sagittal advancement (*r* = −0.338, *p* = 0.001). These correlations indicate that larger sagittal advancement at A, Sd, and Id was associated with more soft-tissue thinning in the corresponding regions (Fig. 4). No other sagittal correlations were significant. The ratio between soft-tissue thickness change and sagittal skeletal advancement is shown in Fig. 3B.

For vertical skeletal movements (Table 8), B-sl thickness change was positively correlated with Id and point B downward vertical movement, whereas Me-Me’ thickness change was negatively correlated with Id, point B, Pog, and Me vertical movement. No other vertical correlations were significant.

## Discussion

The present study evaluated regional soft-tissue thickness changes after bimaxillary orthognathic surgery in skeletal Class II patients without genioplasty. The main finding was a general postoperative thinning tendency in the anterior facial soft-tissue, especially at Id-li, followed by Pog-Pog’, A-sn, and B-sl. The ratio of soft-tissue thickness change to skeletal sagittal advancement was highest at Id-li/Id (0.30), followed by A-sn/A (0.20), Sd-ls/Sd (0.08), Pog-Pog’/Pog (0.07), B-sl/B (0.05), and Me-Me’/Me (0.03). These findings are consistent with recent 3D evidence showing that soft-tissue change after orthognathic surgery vary by region and are not fully explained by skeletal movement alone [9, 20].

Although the sex distribution was not statistically balanced, with 56 females and 33 males among the 89 patients, this cohort was less female-dominant than comparable studies, including Storms et al. (77 females, 32 males), Gasimov et al. (42 females, 18 males), and Seon et al. (40 females, 10 males) [7, 21, 22]. Moreover, sex was not significantly correlated with any regional thickness change, suggesting that the main findings were unlikely to be driven by sex imbalance.

A notable feature of this study is the relatively long follow-up interval. The mean CBCT interval was 16.7 ± 5.3 months, whereas many previous studies used short-term postoperative records. Early postoperative CBCT may be useful for evaluating surgical skeletal position, but it is less suitable for soft-tissue thickness assessment because postoperative edema can still influence the measurements. Quantitative swelling studies indicate that facial edema decreases rapidly in the early postoperative period but may continue to change over several months [23, 24]. Therefore, the present follow-up window reduces the influence of early swelling and better reflects the combined final effect of surgery, postsurgical orthodontic finishing, and soft-tissue adaptation [25]. Given the multifactorial nature of postoperative soft-tissue adaptation, reductions in hard-to-soft tissue thickness should be interpreted as morphometric changes that may reflect alterations in soft-tissue posture, contour remodeling, tissue tension, or lip configuration, rather than direct evidence of regional soft-tissue volume loss.

Id-li showed the most pronounced thinning (2.20 ± 2.11 mm), and larger sagittal advancement at Id was associated with more thinning. Because Id advanced similarly to B, Pog, and Me, its sagittal displacement was considered to mainly reflect mandibular advancement rather than postoperative orthodontic incisor movement. This mandibular advancement may have induced backward rotation or flattening of the lower lip, as reflected by the concomitant increase in the labiomental angle, thereby contributing to the observed lower-lip thinning. Storms et al. similarly reported lower-lip thinning after mandibular advancement in Class II patients, with lower-lip movement representing only 76% of the corresponding hard-tissue movement [7]. In the present study, the Id-li/Id ratio was approximately 30%, lower than several landmark-displacement ratios reported previously [7, 8]. This may reflect the longer follow-up and the fact that this study measured soft-tissue thickness rather than only soft tissue landmark displacements. Clinically, this suggests that lower-lip and chin soft tissues do not simply translate with the mandible, and that the effect of soft-tissue thinning on the final soft tissue facial profile should be anticipated when planning large mandibular advancements. The same concern is consistent with current interest in improving soft-tissue prediction and, in selected patients, adjunctive soft-tissue volume procedures such as fat grafting [30, 31, 32].

In the lower lip-chin complex, B-sl and Pog-Pog’ showed smaller but significant thinning (0.33 ± 1.30 mm and 0.48 ± 1.29 mm, respectively), but neither was significantly associated with sagittal skeletal advancement. B-sl thickness change was positively correlated with the downward vertical movement of both point B and Id, indicating that larger downward movement of the mandibular dentoalveolar region was associated with less thinning at the sublabiale region. This finding may be explained by opening and flattening of the labiomental fold after anteroinferior mandibular repositioning. Sublabiale represents the deepest point of the labiomental fold, and previous studies have shown that labiomental fold morphology is influenced by vertical chin/lower-face changes and that increasing the labiomental angle may open the fold [26, 27]. In the present study, the labiomental angle increased significantly by 23.43 ± 16.73°, supporting this interpretation. Therefore, larger downward vertical movement of B and Id may increase the outward tension and contour remodeling around the labiomental sulcus, partially preserving B-sl thickness, whereas Pog-Pog’ thickness change may reflect broader adaptation of the chin soft tissue. Although the Me-Me’ thickness change was small and not statistically significant (0.20 ± 1.38 mm, *p* = 0.165), larger downward vertical movement of Me and higher BMI were associated with more Me-Me’ thickness reduction. This suggests that Me-Me’ thinning may be more apparent in patients with larger downward vertical movement of Me and higher BMI.

A-sn thickness also decreased significantly and was associated with sagittal displacement of A and Sd, suggesting that maxillary advancement may increase upper-lip soft-tissue tension and lead to adaptive thinning, consistent with previous evidence that maxillary movement affects nasolabial soft tissues [21, 28].

Transverse skeletal changes were not emphasized because maxillary transverse displacement was small and not significant at A and Sd; in this coordinate system, transverse movement represents left-right displacement relative to the midsagittal plane and most likely reflects minor midline positional adjustment related to pre- and postoperative orthodontic dental midline correction. Thus, sagittal and vertical movements were more interpretable predictors of thickness change in this cohort.

The angular findings further support clinical interpretation. The nasolabial angle did not change significantly, consistent with previous bimaxillary soft-tissue studies, whereas the labiomental angle increased markedly from 109.73 ± 20.62 degrees to 133.16 ± 11.40 degrees [4, 21]. This likely reflects forward and downward mandibular repositioning, reduced labiomental fold depth, and thinning of the lower lip/chin complex. Importantly, because genioplasty was excluded, the result suggests that selected skeletal Class II patients can obtain substantial labiomental profile improvement through bimaxillary surgery alone.

No significant associations were found between soft-tissue thickness changes and age, sex, preoperative or CBCT interval. This suggests that, within the present sample and follow-up range, skeletal movement is a more important determinant of regional thickness change than these patient-related variables. Nevertheless, BMI deserves further study. In the present study, BMI was analyzed as a preoperative baseline patient characteristic, and no longitudinal postoperative BMI data were available. Therefore, postoperative changes in body weight or muscle mass could not be assessed or accounted for in the analyses. Gasimov et al. found that BMI and muscle mass decreased after bimaxillary orthognathic surgery, with the greatest reduction at 1 month and incomplete recovery by 6 months [22]. A postoperative decrease in body weight or muscle mass could theoretically contribute to facial soft-tissue thinning and may therefore represent an unmeasured confounding factor when interpreting the observed associations involving BMI. Future prospective studies should combine CBCT or stereophotogrammetric soft-tissue analysis with serial BMI and orthodontic movement data.

Although CBCT is primarily designed for hard-tissue imaging and provides limited soft-tissue contrast compared with magnetic resonance imaging (MRI) and 3D stereophotogrammetry, it can reliably be used to obtain linear hard-to-soft tissue thickness measurements when standardized anatomical landmarks are used [14]. Moreover, the high spatial resolution of CBCT enables accurate visualization of skeletal and dentoalveolar landmarks, which facilitates consistent landmark identification and improves measurement reproducibility, particularly in longitudinal studies involving pre- and postoperative comparisons. Because CBCT is routinely acquired for orthognathic diagnosis and treatment planning, an increasing number of studies have utilized CBCT-derived soft-tissue measurements to investigate postoperative facial soft-tissue changes [2, 9, 17]. Furthermore, a recent study comparing MRI- and CBCT-derived facial soft-tissue thickness measurements provided further evidence supporting the feasibility and clinical applicability of CBCT-derived region-specific soft-tissue thickness assessment in craniofacial research [29].

Several limitations should be acknowledged. First, the retrospective design means that postoperative CBCT timing depended on clinical indications rather than a fixed research protocol. Second, detailed information regarding postsurgical orthodontic treatment was not consistently available. Therefore, its potential influence on postoperative soft-tissue outcomes should be considered a study limitation and a potential confounding factor. Third, although CBCT-based measurements were performed in three dimensions, soft-tissue outcomes were limited to linear thickness measurements rather than volumetric analyses. Therefore, the study assessed regional soft-tissue thickness changes but could not fully characterize volumetric remodeling of the lips, chin, and perioral tissues. Decreases in linear hard-to-soft tissue thickness should thus be interpreted cautiously, as they may reflect changes in soft-tissue posture, tension, contour adaptation, or lip configuration after skeletal repositioning rather than true tissue volume loss. Fourth, because all patients underwent bimaxillary surgery without genioplasty, the study population may represent a selected subgroup of skeletal Class II patients. Therefore, the findings should not be generalized to patients with more severe chin deficiencies requiring concomitant genioplasty, nor to single-jaw surgery or skeletal Class III deformity, without further validation. Finally, because multiple pairwise Pearson correlations were tested without adjustment for multiple comparisons, statistically significant associations should be interpreted as exploratory rather than confirmatory.

## Conclusion

In skeletal Class II patients treated with bimaxillary orthognathic surgery without genioplasty, anterior facial soft-tissues thickness tended to decrease after treatment. The most significant thinning occurred at Id-li, and this change was significantly related to sagittal advancement of Id, with an Id-li/Id ratio of 0.30. A-sn, B-sl, and Pog-Pog’ also showed significant thinning, whereas Sd-ls and Me-Me’ did not change significantly. Soft-tissue thickness changes were not significantly associated with sex, age, preoperative BMI, or follow-up interval. These findings suggest that postoperative soft-tissue thinning should be considered when predicting the final facial profile after orthodontic-orthognathic correction in soft-tissue-oriented treatment planning.

## Supplementary Information

Below is the link to the electronic supplementary material.

Supplementary File 1 (DOCX 32.6 KB)

Supplementary File 2 (DOCX 32.0 KB)

## Data Availability

No datasets were generated or analysed during the current study.
