# Supplementary material for: Soft-tissue thickness changes following bimaxillary surgery in skeletal class II malocclusion
Source: Clin Oral Investig. 2026 Aug 11;30(9):383. doi: 10.1007/s00784-026-07074-5 (PMC13457495; doi:10.1007/s00784-026-07074-5)
Supplement: Supplementary file 2 [file 784_2026_7074_MOESM2_ESM.docx]

**Supplementary Table 1.** Duplicate measurement error (DME) of the soft-tissue thickness and angular measurements at T0 and T1.

| **Variable** | **DME T0** | **DME T1** |
| --- | --- | --- |
| A_sn (mm) | 0.30 | 0.22 |
| Sd_ls (mm) | 0.25 | 0.14 |
| Id_li (mm) | 0.33 | 0.31 |
| B_sl (mm) | 0.20 | 0.17 |
| Pog_Pog' (mm) | 0.18 | 0.16 |
| Me_Me' (mm) | 0.19 | 0.35 |
| LMA (degree) | 0.99 | 1.41 |
| NLA (degree) | 1.22 | 0.86 |
